# Assessing Potential Vulnerability and Response of Fish to Simulated Avian Predation after Exposure to Psychotropic Pharmaceuticals

**DOI:** 10.3390/toxics4020009

**Published:** 2016-04-13

**Authors:** Melanie L. Hedgespeth, Per Anders Nilsson, Olof Berglund

**Affiliations:** 1 Aquatic Ecology, Department of Biology, Lund University, Sölvegatan 37, 223 62 Lund, Sweden; anders.nilsson@biol.lu.se (P.A.N.); olof.berglund@biol.lu.se (O.B.); 2 Department of Environmental and Life Sciences—Biology, Karlstad University, 651 88 Karlstad, Sweden

**Keywords:** sertraline, fluoxetine, propranolol, SSRI, beta-blocker, fish, bird, behavior, predation

## Abstract

Psychotropic pharmaceuticals present in the environment may impact organisms both directly and via interaction strengths with other organisms, including predators; therefore, this study examined the potential effects of pharmaceuticals on behavioral responses of fish to avian predators. Wild-caught juvenile perch (*Perca fluviatilis*) were assayed using a striking bird model after a seven-day exposure to psychotropic pharmaceuticals (the antidepressants fluoxetine or sertraline, or the β-blocker propranolol) under the hypotheses that exposure would increase vulnerability to avian predation via increasing the probability of predator encounter as well as degrading evasive behaviors upon encounter. None of the substances significantly affected swimming activity of the fish, nor did they increase vulnerability by affecting encounter probability or evasive endpoints compared to control treatments. Counter to our expectations, fish exposed to 100 μg/L fluoxetine (but no other concentrations or pharmaceuticals) were less likely to enter the open area of the arena, *i.e.*, less likely to engage in risky behavior that could lead to predator encounters. Additionally, all fish exposed to environmentally relevant, low concentrations of sertraline (0.12 μg/L) and propranolol (0.1 μg/L) sought refuge after the simulated attack. Our unexpected results warrant further research as they have interesting implications on how these psychotropic pharmaceuticals may affect predator-prey interactions spanning the terrestrial-aquatic interface.

## 1. Introduction

Due to their presence in aquatic environments and biota, pharmaceuticals designed to modulate human behavior have the potential to mediate behavioral changes in non-target organisms, as examined in studies on psychotropic groups including antidepressants and anxiolytics using fish [1,2,3,4,5]. These effects likely occur because many features of the nervous system involved in the regulation of behavior within animals are largely evolutionarily conserved across taxa [6], including potential drug targets [7]. In this sense, psychotropic pharmaceuticals are of particular interest because they are designed to target the nervous system in human consumers with pharmacological effects on mood, cognition, and behavior. Psychotropic pharmaceuticals in the environment may therefore impact behaviors of exposed organisms including their interactions with the surrounding biotic and abiotic environment. Accordingly, studies have recently indicated that behavioral changes induced by pharmaceutical exposure may result in effects not only on the Darwinian fitness of directly exposed individuals, but that changes in behavior have implications for effects at larger ecological scales including populations and communities [8,9,10].

Organism interactions link individual-level effects on behavior to broader ecological responses, especially those spanning multiple trophic levels such as the interactions involved in encounters between predators and their prey. In terms of predator-prey interactions and predation risk, aspects of behavior that may be affected by pharmaceuticals include those that influence components of the predation sequence: the rate of encounter between predators and prey, the probability of death due to an encounter, and time spent vulnerable to an encounter [11,12]. Moreover, prey organisms including fish face behavioral tradeoffs due to the amount of energy and time that can be allocated to vigilance (e.g., avoiding predation risk) *versus* food acquisition or reproduction [13]. Chemically-induced behavioral changes may therefore affect this allocation, influencing encounter rates with predators and, additionally, impacting the immediate behavioral responses of prey to encounters important for evasion success.

An important consideration regarding trophic interactions is that although contaminants may equally impact organisms from all trophic levels, it is more likely that contaminants differentially affect various species due to intra- and interspecific differences in traits (e.g., physiology, life history, habitat choice, *etc.*), affecting both susceptibility and sensitivity to exposure [14]. In such cases, one may expect that a “trophic sensitivity mismatch” to environmental contaminants could result in complex consequences for aquatic community dynamics. As a result, the direct effects upon predators may indirectly affect their prey through altered predation pressure or, conversely, direct effects on prey may subsequently impact the predator due to altered resource availability. In this sense, behavioral effects on fish exposed to pharmaceuticals may result in indirect impacts that are limited not only to the aquatic environment, but may, for example, also affect feeding success of piscivorous avian predators not subjected to exposure via water. Fish experience direct uptake from water and uptake via feeding, whereas birds are exposed only via feeding [15,16]. Fish prey could therefore be adversely affected by pharmaceuticals causing changes in behavior, resulting in their increased predation susceptibility to relatively non-impacted predators.

The interactions and levels of the neurotransmitters serotonin, dopamine, and GABA are key in modulating behavioral traits in humans, mammals, and birds [17], and there is recent evidence for such modulation in fish as well (reviewed by [18]). Additionally, neurotransmitters such as adrenaline are vital in the short-term behavioral responses of the fight-or-flight response and in the expression of behaviors associated with aggression and anxiety [18,19]. Therefore, groups of psychotropic pharmaceuticals targeting these systems and with potential impacts on anti-predator behaviors of non-target organisms are of particular interest to our study.

Antidepressant selective serotonin reuptake inhibitors (SSRIs) such as fluoxetine and sertraline are used in the treatment of depression and other mood disorders by binding serotonin receptors and blocking the reuptake of serotonin in nerve synapses. Not only have these chemicals been detected in environmental water samples and fish tissues, but multiple studies have detected behavioral effects of SSRIs in fish (reviewed by [20,21]). Another pharmaceutical class commonly detected in surface waters serving as wastewater recipients, the β-blockers, also has implications for toxic effects in fish (reviewed by [22,23]). In humans, β-blockers such as propranolol act via blocking the stimulation of adrenaline and noradrenaline on the β1- and β2-adrenergic receptors fundamental for initiating the “fight-or-flight” response. Though β-blockers are primarily used in the treatment of cardiovascular disorders, propranolol can enter the central nervous system and has also been used to treat anxiety. The β-adrenergic receptors have been previously identified in fish and regulate multiple physiological systems including cardiac function. Though propranolol exposure in the low μg/L range has been shown to influence the heart rate in fish embryos [24], reports of effects on behavior in fish are scarce (but see [25]).

Here, we tested the potential behavioral effects of pharmaceuticals on fish in terms of impacts on vulnerability to avian predation, which to our knowledge has not yet been addressed in the current scientific literature. We did so by behaviorally assaying wild-caught, juvenile perch (*Perca fluviatilis*) after exposing them to one of three psychotropic pharmaceuticals (the SSRIs fluoxetine or sertraline, or the β-blocker propranolol). We expected that exposure to the SSRIs or β-blocker might impact the fish by increasing their vulnerability to avian predation via increasing the probability of encounters, as well as negatively impacting subsequent evasive behaviors after a simulated predator encounter. Endpoints of interest included general swimming activity of fish and willingness to feed in a novel environment (*i.e.*, making the fish susceptible to predation by placing them in an open, uncovered area of the arena), after which we simulated an encounter with an avian predator using a striking bird model to assess initial escape velocity, the use of cover, and time taken to reach cover, post-strike.

## 2. Materials and Methods

### 2.1. Animal Collection

Juvenile Eurasian perch (*Perca fluviatilis*, 7.5 cm ± 3 cm) were collected from Lake Krankesjön in southern Sweden in summer–autumn 2014, on which the impact by wastewater treatment facilities is negligible to none. Fish were allowed to acclimate (12:12 h l:d cycle at 12 °C room temperature) in flow-through tanks and were fed frozen mosquito larvae and live *Daphnia magna* during the acclimation period (≥one week). Experiments were carried out according to ethical approval no. M459-12, Malmö/Lund djurförsöksetiska nämnd, Lund, Sweden.

### 2.2. Chemical Exposures

Sertraline HCl (CAS# 79559-97-0, Toronto Research Chemicals Inc., Toronto, ON, Canada) was dissolved in dimethyl sulfoxide (DMSO, Sigma Aldrich, St. Louis, MO, USA) and spiked into 3.5 L dechlorinated tap water, resulting in nominal exposure concentrations of 0.12, 89, or 300 μg/L (based on prior experiments [10]). Fluoxetine HCl (CAS# 59333-67-4, Toronto Research Chemicals Inc.) and propranolol HCl (CAS# 318-98-9, Sigma Aldrich) stocks were also prepared separately by dissolution in DMSO and spiked into 3.5 L dechlorinated tap water, resulting in nominal exposure concentrations of 0.1, 1, or 100 μg/L. These concentrations of fluoxetine and propranolol were chosen in order to represent high environmental concentrations (based on surface water, wastewater effluent, and predicted environmental concentrations) up to those shown to result in effects on fish [26,27,28,29], or where the fish plasma model [30] would predict concentrations above human therapeutic levels (C_max_). Two blocks of experimental trials were run for each chemical’s treatment combination: one trial consisted of exposure to a solvent control treatment (sertraline 0.0018% *v*:*v* DMSO:H_2_O; fluoxetine and propranolol 0.001% *v*:*v* DMSO:H_2_O), along with the remaining three treatment concentrations per chemical (*n* = 5 fish per chemical treatment per trial, resulting in a total of *n* = 10 fish per concentration for each of the three chemicals). Fish were exposed individually in 5 L glass containers for between seven and nine days per trial (further described in Section 2.3). This exposure time was chosen for the SSRIs as no abiotic degradation would be expected to occur [31], it is sufficient to reach steady state between fish and water (*i.e.*, within four to seven days [31]), and behavioral effects have been detected after this exposure time in other studies [10,32]. Regarding propranolol, no abiotic degradation would be expected to occur during this exposure time [33], though literature data related to steady state and behavioral effects in fish are scarce (but [25] detected behavioral effects after a two-week exposure period). Fish were fed frozen mosquito larvae every other day during exposure and were not fed <24 h prior to behavioral assays. Behavioral assays were run on days 7, 8, and/or 9 of exposure, after which trials were terminated and fish were measured for body length. Exposure medium pH was measured at 8.1 ± 0.2 throughout the exposure periods for all trials.

### 2.3. Behavioral Assays

Circular arenas for the behavioral assays were made of PVC (60 cm diameter), the bottom of which was marked with a circular strike zone (10 cm diameter) with its center 20 cm from the closest edge of the arena. A refuge area (15 cm × 20 cm) consisting of a small PVC tube and artificial macrophyte was located at the opposite end. Arenas were filled to a 9 cm depth with dechlorinated tap water, which was renewed prior to each individual assay. A fine mesh bag containing thawed mosquito larvae was placed at the center of the strike zone prior to each assay and this was renewed every other assay.

After seven days of exposure to the chemical treatments in an experimental trial, individuals were placed into the refuge area of the arena and given 20 min maximum to enter the strike zone. When more than half of the fish’s body length entered the strike zone, a plastic bird model was released, striking the surface of the water directly over the center of the strike zone and then immediately retracted. A Logitech HD Pro 920 webcam (Logitech, Newark, CA, USA) was positioned centrally above the arena and connected to a computer for remote viewing and recording of assays to avoid disturbance to the fish. All videos were recorded at 30 frames per second beginning with the addition of the fish to the arena until 5 min after the release of the bird model. All fish from an experimental trial were assayed after 7 d of exposure; those that did not enter the strike zone within the 20 min cap were placed back into their respective exposure containers and the assay was repeated on day 8. Due to low sample size for some trials (*n* < 10), we assayed fish again on day 9: fluoxetine trial 1 and propranolol trials 1 and 2. If fish did not enter the strike zone (initiate the assay) on any of the assayed days, the individual was scored as “0” for entering the strike zone (*i.e.*, binomial response) with a time of “NA”.

### 2.4. Video and Data Analyses

Endpoints measured for all chemicals from the behavioral assays were: (1) general swimming activity of fish; (2) proportion of fish entering the strike zone, and for those that did; (3) initial escape velocity after release of the striking bird model; (4) proportion of fish entering cover after the strike, and for those that did; (5) time taken to reach cover. Endpoints were assessed using Windows Movie Maker (Microsoft Corporation, Redmond, WA, USA, v. 2012) and were scored blindly for all videos prior to statistical analyses. Endpoints for each pharmaceutical were scored separately, for which the data from each chemical’s two respective trials were analyzed together, assessing endpoint (DV) as an effect of treatment concentration (IV) in all statistical models. This was done separately for each chemical. The cutoff for statistical significance was set at α = 0.05 for all analyses.

General swimming activity (endpoint 1) was measured as the time fish spent moving during the behavioral assay normalized to total assay time to give a percentage value. This was measured from the recorded videos using a stop-watch, for which time in motion was defined as active locomotion transporting the fish >10% of its body length per second [34]. Fish not entering the strike zone were observed for the full 20 min period of the assay and fish entering the strike zone were observed until the release of the bird model (*i.e.*, directly prior to initiation of an escape response).

The initial escape velocity of each fish (endpoint 3) was measured as the snout-to-snout distance traveled within the first 0.1 s (three frames) after the initiation of an evasive maneuver (c-start response) using ImageJ (v. 1.47 g, National Institutes of Health, Bethesda, MD, USA). Initial escape velocity was then normalized to the fish’s body length (BL/s); none of the fish changed direction from their initial escape trajectories within the 0.1 s timeframe.

For analysis of the proportion of fish entering cover post-strike (endpoint 4), videos were viewed frame-by-frame for the first 2 s (60 frames) after the fish initiated an evasive maneuver. Fish were scored as entering cover if fish swam to the refuge area or the edge of the arena and remained in cover for the remainder of the 2 s timeframe (within a distance of 0.5 body lengths).

Data were analyzed in R [35]. For endpoint 1, activity of fish that did not enter the strike zone was significantly less than those that did for all chemicals (compared for each chemical separately and pooled across concentrations using a Wilcoxon/Mann–Whitney rank sum test). Therefore, this endpoint was analyzed separately for the two groups of fish as a function of each chemical’s treatment concentration using an analysis of variance (ANOVA) with log transformation, or subsequently a Kruskal-Wallis Rank Sum Test if data did not meet parametric test assumptions. Endpoints 2 and 4 were analyzed via GLM (generalized linear model) assuming a binomial distribution with a logit link. Model fits were checked by examining plots of residuals, AIC scores, and residual deviance. For endpoints 3 and 5, fish that did not enter the strike zone (*i.e.*, scored as “0” for endpoint 2) were removed from analyses, and for endpoint 5, fish that did not seek cover were removed from the analysis. Only one fish initiated a “freeze” response during the entire experiment (*i.e.*, no initiation of evasive maneuver post-strike; in sertraline trial 2) and was therefore removed from subsequent analyses of endpoints 3–5. The remaining data were analyzed via ANOVA with log transformation if necessary to meet the assumptions of normality and homoscedasticity.

## 3. Results

**Figure 1:**
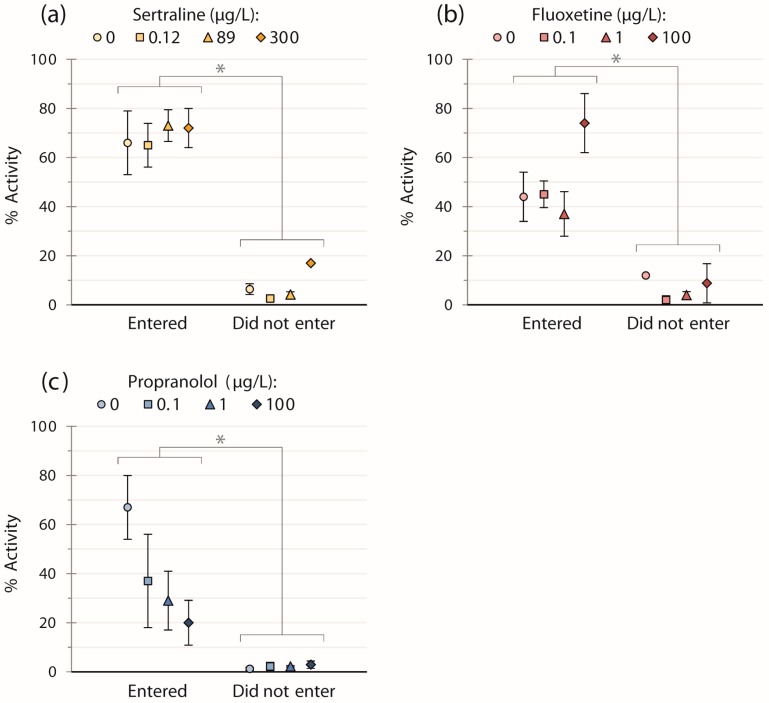
Means ± SEM of the general swimming activity of fish exposed to: (**a**) sertraline (*n* = 39); (**b**) fluoxetine (*n* = 40); or (**c**) propranolol (*n* = 39), for fish that entered and did not enter the strike zone. Fish entering the strike zone displayed higher activity *versus* those which did not (*p* < 0.001 for all three pharmaceuticals, regardless of concentration). There were no significant effects of pharmaceutical concentration within the two groups.

For all three chemicals, the general swimming activity of fish was significantly less by factors of 12 (sertraline trials), eight (fluoxetine trials), and 18 (propranolol trials) for those fish not entering the strike zone *vs.* those that did regardless of concentration (Figure 1; sertraline: *W* = 0, *p* < 0.001; fluoxetine: *W* = 18, *p* < 0.001; propranolol: *W* = 9, *p* < 0.001). However, we did not detect any significant effects of chemical concentration on activity when these two groups were analyzed separately per chemical (Figure 1).

We did not detect effects of sertraline or propranolol treatments on the proportion of fish entering the strike zone (56%–80% and 40%–70%, respectively); however, there were statistically significant effects of fluoxetine treatment (Table 1). Fish in the 100 μg/L treatment had a lower probability of entering the strike zone (40% ± 15% SEM) compared to those in the control treatment (90% ± 9.5% SEM) based on predicted model values (0 *vs.* 100 μg/L GLM comparison: *z* = −2.11, *p* = 0.035; model AIC = 52.4, residual deviance = 44.4, *df* = 36).

**Figure 2:**
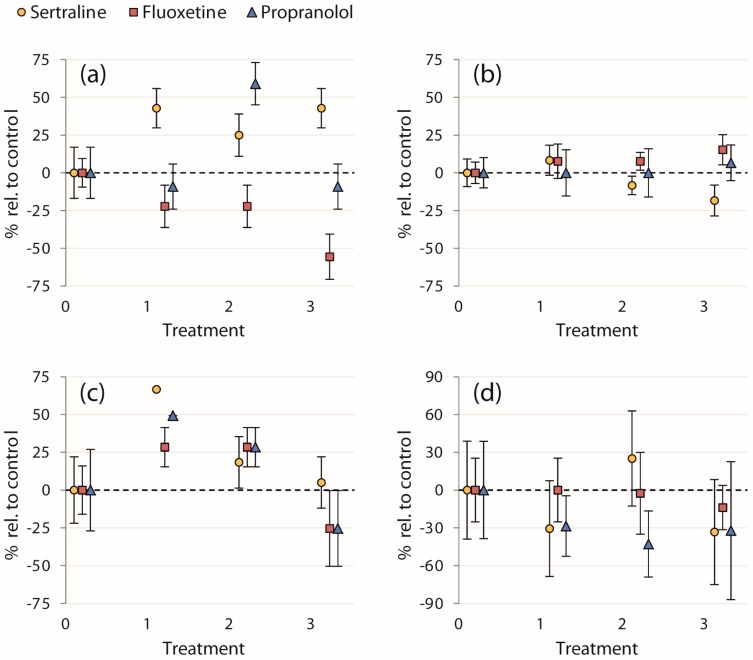
Calculated mean percent changes in endpoints relative to the control treatments ((treatment mean − control mean)/control mean × 100) ± SEM (% of each treatment mean): (**a**) proportion of fish entering the strike zone; (**b**) initial escape velocity after the strike; (**c**) proportion of fish entering cover after the strike; and (**d**) time taken to reach cover. Treatments on the *x*-axis are **0**: control; **1**: 0.12 and 0.1 μg/L; **2**: 89 and 1 μg/L; and **3**: 300 and 100 μg/L for sertraline and fluoxetine/propranolol, respectively.

Our study did not reveal statistically significant effects of any of the chemical treatments on evasive responses, *i.e.*, neither on the initial escape velocities of fish (ranging from 9.8–16 BL/s), nor on the proportions of fish entering cover and concurrent time taken to reach cover (results for all endpoints are presented in Table 1). Though the effects of treatment on proportions of fish entering cover were not statistically significant, 100% of fish in the 0.12 μg/L and 0.1 μg/L sertraline and propranolol treatments, respectively, entered cover after the strike—a 67% and 49% increase, respectively, relative to the control treatments (Figure 2c). Mortality was low for the entirety of the experiment (*n* = 1, in the control treatment of propranolol trial 2).

## 4. Discussion

The behavioral endpoints assayed in this study represent some of the key components involved in the sequence of events of predation risk [12] that occur during encounters between aquatic prey and avian predators. The entry of the fish into the strike zone places it in an open area of the arena with the possibility of obtaining food; however, this also increases its vulnerability to encounters with avian predators, as represented by the striking bird model in our assay. Subsequently, the fish’s ability to escape the simulated predator encounter was assayed in subcomponents: via measuring its initial escape velocity as well as whether/how quickly the fish seeks cover, *i.e.*, refuge from predation.

Fluoxetine was the only pharmaceutical that elicited a statistically significant effect on fish behavior in this study (Table 1, Figure 2a). Exposure to the highest concentration of fluoxetine at 100 μg/L reduced the probability of entering the strike zone by 56% in comparison to the control treatment. On average, the activity of fish not entering the strike zone was significantly lower than that of those that did for all chemicals; interestingly, however, the general swimming activity of the fish in these two groups was not significantly affected by exposure to fluoxetine (Figure 1b). Another study noted reductions in locomotor activity of juvenile fish after exposure to 300 μg/L fluoxetine [36], three times higher than the highest concentration used in our study. These results indicate that factors other than swimming activity (e.g., effect on feeding motivation or exploratory behavior) may have played a role in the reduced propensity of fish to enter the strike zone after exposure to fluoxetine.

Entry into the strike zone may be an indirect measure of a fish’s interest in the food item and/or willingness to feed in an open, novel environment (the arena). Such risk-taking behavior involves an important trade-off between the animal’s need to acquire food and the amount of vigilance that it can devote to potential risks present in novel and/or open environments (e.g., predation). As a result, behavioral studies have utilized the propensity of zebrafish to inspect a novel object [37] and the willingness of perch to feed in an open environment based upon perceived predation risk [38] as measures of boldness along the shy-bold continuum of animal behavioral traits [39], a key aspect of animal personality and behavioral syndromes [40]. Though this effect did not occur at concentrations under 100 μg/L in our study, this is in agreement with a recent study that found decreased boldness of fish (*Betta splendens*) exposed to fluoxetine at 0.5 and 5 μg/L for eight days compared to control fish [41]. Because our experiment compared treatment means instead of repeated measures on individuals, we cannot definitively conclude that there were changes within an individual in terms of its boldness due to chemical exposure; however, the behavioral decisions involved imply that the fish exposed to the highest fluoxetine treatment were “shyer” than control fish, on average, by engaging in less risky behavior, and potentially at the cost of reduced food intake.

By controlling for hunger prior to behavioral assays (not feeding <24 h prior to assays), we assumed fish would be attracted by the mesh food bag placed into the center of the strike zone due to the diffusion of mosquito larvae cues in the water. However, fluoxetine exposure within a similar concentration range and duration has been shown to affect feeding in fish in other studies: Stanley and coauthors [42] reported decreased feeding rates of juvenile fathead minnows on zooplankton, and Gaworecki and Klaine [32] reported increased capture times with a resulting decrease in the amount of live prey eaten by adult hybrid striped bass. Whether the reduction in the proportion of fish entering the strike zone after fluoxetine exposure in the current study was motivated via a similar process, *i.e.*, possibly by reduction in appetite mediated by serotonin, remains to be determined.

Interestingly, sertraline exposure did not appear to impact swimming activity or willingness to feed in a novel environment in the present study. Xie *et al.* [43] found that a seven-day exposure to sertraline (4.36–116 μg/L) resulted in an increase in swimming activity, reduced shoaling tendency, and reduced feeding rate/food consumption of carp. We have also previously shown reduced feeding rates of juvenile perch on zooplankton at multiple prey densities (fish were collected from the same lake and exposed to the same sertraline concentrations as the present study; [10]). Although both fluoxetine and sertraline act via the same mechanism of action in humans (by blocking serotonin receptors, reducing the reuptake of serotonin from nerve synapses), pharmacological data on SSRIs indicate that fluoxetine binds less selectively to the serotonin receptor with increased potential for interaction with dopamine and norepinephrine receptors, leading to increased levels of those neurotransmitters [44]. Additionally, fluoxetine is metabolized into an equally effective metabolite, norfluoxetine, as compared to sertraline’s inactive, primary metabolite [45,46]. These factors may explain fluoxetine’s apparent higher efficacy on the willingness to feed in a novel environment compared to sertraline observed in our study.

Exposure of fish larvae to SSRIs has been shown to result in alterations in c-start response in a previous study [47]. Fluoxetine exposure (12 d, 250 ng/L) resulted in a slower escape velocity, though exposure to sertraline alone did not appear to affect behavior, and exposure to a mixture of SSRIs (fluoxetine, sertraline, venlafaxine, and bupropion) resulted in slower escape velocity and overall diminished escape response. Another study has shown that after four-week exposure of adult fathead minnows to 1–100 μg/L fluoxetine, fish reduced the distance swum after being startled by the visual stimulus of a mock predator, and some of those in the highest treatments did not appear to respond at all [48]. Although we did not study some of these specific endpoints directly, there was no evidence of effects of either of the two SSRIs on predator evasion in our study.

Domenici [49] describes the importance of considering context-dependence of the anti-predator responses of fish for which factors such as the presence of food, distance to refuge, stimulus strength, *etc.*, appear to play a large role. Not only were the fish in the present study exposed for a shorter duration (compared to [48]), but the stimulus used was also stronger, resulting in both visual and mechanical disturbance of the water surface directly above the fish. Additional studies of SSRIs on behavior indicate increased susceptibility to predation after exposure: juvenile fish injected with 10 μg/g fluoxetine did not respond to conspecific alarm cues [1], tadpoles exposed to 3 μg/L fluoxetine for three weeks did not hide in the presence of predator cues [50], and the exposure of adult fish to sertraline (28 d, 3–30 μg/L) reduced time spent in shelters [5]. Interestingly, we also made a similar qualitative observation as Gaworecki and Klaine [32]: that some of the fish exposed to 100 μg/L fluoxetine maintained a vertical position in both the exposure container and behavioral arena. Though this may have implications for visual detection by and, subsequently, encounters with avian predators, it did not appear to affect the evasive response of fish in our assay.

We expected that after exposure to propranolol, fish would demonstrate behavioral changes in response to the strike by the simulated predator, *i.e.*, effects on the fight-or-flight response; however, we did not detect any effects on the behaviors assayed in our study. Though not statistically significant, there was a trend for lower swimming activity with increasing concentrations of propranolol for those fish entering the strike zone (Figure 1c); further studies into the potential for effects on activity may be warranted. To our knowledge, no studies to date have reported behavioral endpoints regarding predation risk of fish after propranolol exposure. However, a recent study on adult zebrafish has shown that exposure to 0.6 μg/L increased the number of transitions fish made to the upper half of a novel tank environment, and fish exposed to 6 mg/L spent significantly more time in the upper portion of the tank compared with controls, both being indicative of lower anxiety [25]. Physiological effects have also been demonstrated to occur at high concentrations of propranolol in water (mg/L range) or after injection [27,51] and inconsistent results have been reported for reproductive endpoints [52,53]. One study reported effects of propranolol on the reproductive behavior in male fish, for which males exposed to nominal concentrations of 0.1 μg/L and 1 μg/L visited nests less frequently than controls, though this effect did not occur at 10 μg/L [3].

Given that the drug targets of the three compounds in this study are present in both fish and humans, we may hypothesize effects on perch at human therapeutic levels [23,46]. The fish plasma model has been suggested as a tool to predict pharmaceutical effect levels in aquatic organisms induced by similar modes of action as in humans [30]. Assuming blood:water partitioning coefficients according to the model presented by Fitzsimmons and colleagues [54] and human therapeutic plasma concentrations (C_max_) of fluoxetine, sertraline and propranolol, plasma exposure ratios are >1 for the highest treatment concentrations in this study, *i.e.*, theoretical fish plasma levels are above human therapeutic levels. Thus, the nominal concentrations used here would result in fish plasma concentrations in the range of therapeutic mammalian concentrations, and influence on these neurotransmitter systems could be expected. Earlier studies in our laboratory on the uptake of fluoxetine and sertraline in a similar-sized fish, the nine-spined stickleback (*Pungitius pungitius*), indicated that steady-state concentrations were reached within four to seven days and bioaccumulation factors were in the range of 40–50, with experimental half-lives in aquaria containing fish of six and nine days for sertraline and fluoxetine, respectively [31]. In addition, there was no significant abiotic degradation of either fluoxetine or sertraline in test media (pH 6.5, 7, or 9) exposed to constant light (995 ± 124 lux) over a 35 d period [31]. Regarding propranolol, literature data indicate that abiotic degradation is low, with an estimated environmental half-life of >1 year [33], and that two species of fish (*Hemiculter leucisculus*, *Carassius auratus*) sampled from the field had bioaccumulation factors in the range of 133–4000 [55]. This information suggests that the absence of observed effects on many of the behavioral endpoints assayed in our study could therefore be due to different drug target sensitivities between humans and this fish species rather than due to differences in uptake kinetics.

Even though almost no statistically significant effects were detected, we suggest that further research on the effects of these pharmaceuticals on the behaviors assayed in this study is warranted. The failure to detect effects in this study may be influenced by the sample sizes and subsequent low statistical power for some of the endpoints, as well as the large variability in the behavior of controls (*i.e.*, among the chemicals). Therefore, we also present the percent change in the mean of each treatment relative to the control mean for a qualitative examination of all four endpoints to evaluate trends in contaminant exposure (Figure 2). Though these normalized data were not analyzed statistically, an interesting trend in the proportion of fish seeking cover after the simulated avian strike was apparent (Figure 2c). Exposure to sertraline and propranolol at the lowest concentrations (0.12 and 0.1 μg/L, respectively) resulted in a large percentage increase in the proportion of fish entering cover relative to the control treatments. All of the fish sought cover in these treatments, whereas this occurred neither in the control nor in higher treatment concentrations (Table 1). Further study is required to determine whether pharmaceutical exposure indeed induced this effect, but if so, this would indicate an enhanced evasive response to avian predation in fish exposed to environmentally relevant concentrations of these two chemicals.

## 5. Conclusions

Concentrations of these three compounds in the range of those found in the environment do not appear to increase the risk for avian predation based on our assays; interestingly, results suggest the opposite—a potential decrease in vulnerability. Not only do the trends in responses of treated fish relative to controls suggest this, but those fish exposed to 100 μg/L fluoxetine were less likely to enter the strike zone. Although this effect is caused by a relatively high concentration of fluoxetine in comparison to that detected in surface water samples, the potential for mixture effects of multiple SSRIs (e.g., [47]) which may also be present in the environment should not be ignored. On average, the fluoxetine-exposed fish may have been “shyer” than those in the control, with a lower propensity to engage in risky behavior that would expose them to either avian or pelagic predators; however, this behavior may make them vulnerable to other piscivores utilizing macrophyte cover, for example “sit-and-wait predators” such as pike (*Esox lucius*; [56]), or wading birds and mammals. Therefore, in terms of assessing the impacts of psychotropic pharmaceuticals on the predation risk of fish, further research on the potential for individual-level changes in personality traits (e.g., boldness) and top-down effects by multiple predators with differing foraging modes is warranted.

## Figures and Tables

**Table 1:** Means ± SEM (*n*) of assayed behavioral endpoints for sertraline, fluoxetine, and propranolol treatments. Values for proportions entering strike zone and cover are predicted GLM means ± SEM. Statistically significant GLM results are presented in bold italics.

Chemical	(μg/L)	Proportion Entering Strike Zone (%)	Initial Escape Velocity (BL/s)	Proportion Entering Cover (%)	Time to Reach Cover (s)
Sertraline	0	56 ± 17 (9)	12 ± 1.1 (5)	60 ± 22 (5)	0.72 ± 0.28 (3)
	0.12	80 ± 13 (10)	13 ± 1.3 (8)	100 ± 0.0020 (8)	0.50 ± 0.19 (8)
	89	70 ± 14 (10)	11 ± 0.67 (7)	71 ± 17 (7)	0.90 ± 0.34 (5)
	300	80 ± 13 (10)	9.8 ± 1.0 (8)	63 ± 17 (8)	0.48 ± 0.20 (5)
Fluoxetine	0	***90 ± 9.5 (10)***	13 ± 0.93 (9)	67 ± 16 (9)	0.79 ± 0.20 (6)
	0.1	***70 ± 14 (10)***	14 ± 1.6 (6)	86 ± 13 (7)	0.79 ± 0.20 (6)
	1	***70 ± 14 (10)***	14 ± 0.83 (7)	86 ± 13 (7)	0.77 ± 0.25 (6)
	100	***40 ± 15 (10)***	15 ± 1.5 (3)	50 ± 25 (4)	0.68 ± 0.12 (2)
Propranolol	0	44 ± 17 (9)	15 ± 1.5 (3)	67 ± 27 (3)	1.4 ± 0.54 (2)
	0.1	40 ± 15 (10)	15 ± 2.3 (3)	100 ± 0.0028 (4)	1.0 ± 0.24 (4)
	1	70 ± 14 (10)	15 ± 2.4 (6)	86 ± 13 (7)	0.80 ± 0.21 (6)
	100	40 ± 15 (10)	16 ± 1.9 (4)	50 ± 25 (4)	0.95 ± 0.52 (2)

## Abbreviations

The following abbreviations are used in this manuscript:

SSRI: selective serotonin reuptake inhibitor
GABA: gamma-aminobutyric acid
C_max_: human therapeutic plasma concentrations
